# The impact of atezolizumab and bevacizumab in hepatocellular carcinoma with activated ß‐catenin signaling

**DOI:** 10.1002/cnr2.1493

**Published:** 2021-07-26

**Authors:** Sebastian Krug, Laura Mattheis, Monika Haemmerle, Jonas Rosendahl, Joerg Kleeff, Patrick Michl

**Affiliations:** ^1^ Department of Internal Medicine I Martin‐Luther University Halle‐Wittenberg Halle (Saale) Germany; ^2^ Institute of Pathology Martin‐Luther University Halle‐Wittenberg Halle (Saale) Germany; ^3^ Department of Visceral, Vascular and Endocrine Surgery Martin‐Luther University Halle‐Wittenberg Halle (Saale) Germany

**Keywords:** angiogenesis, HCC, immunotherapy, response, ß‐catenin

## Abstract

**Background:**

To date, no biomarkers exist to predict response or resistance to immunotherapy in hepatocellular carcinoma (HCC). Recent approaches to classify HCC into different immunological states revealed a negative correlation between Wnt/ß‐catenin activation and immunogenicity and T‐cell infiltration. If these “cold” tumors with primary resistance to checkpoint inhibition (CPI) may benefit from dual treatment of CPI and anti‐angiogenic therapy has not been proved.

**Case:**

Here, we describe the case of a male patient with metastatic HCC. After failure of standard of care treatment with lenvatinib, sorafenib and ramucirumab fourth‐line systemic therapy with atezolizumab and bevacizumab were applied leading to a phenomenal response. Immunohistochemical evaluations were compatible with Wnt/ß‐catenin pathway activation and accompanying low T‐cell infiltration as well as low PD‐L1 score.

**Conclusion:**

Patients with Wnt/ß‐catenin activation may benefit from combination therapy with atezolizumab and bevacizumab regardless of potential predictive markers for immune checkpoint inhibition.

## 1 INTRODUCTION

The hepatocellular carcinoma (HCC) is responsible for an increasing number of cancer‐related deaths worldwide and is usually associated with liver cirrhosis.^1^ Growing evidence indicates metabolic syndrome related to diabetes and obesity as mounting risk factors for HCC in the Western world.^2^ While in patients with liver cirrhosis a multistep progression from cirrhotic nodules to HCC occurs, the mechanism is more heterogeneous in noncirrhotic patients. There, chronic infection with HBV, nonalcoholic steatohepatitis or the malignant transformation of hepatocellular adenoma are involved in cancer development. Preclinically, two major molecular subtypes of HCC exist: a proliferation and nonproliferation class. The latter one is often characterized by an activation of the WNT signaling pathway based on the mutation of *CTNNB1* (encoding for β‐catenin). *CTNNB1* and *TERT* mutations are frequently detected in liver adenomas and are marked by a particularly high rate of malignant progression. Besides tumor cell‐specific alterations the tumor microenvironment (TME) has been increasingly recognized as modulator of tumor initiation and progression. According to the immune status in the TME, the HCC can be classified into three subgroups: immune, immune intermediate, and immune excluded tumors. The immune subtype demonstrated immune cell infiltration and tumors of the immune excluded class showed Wnt/β‐catenin pathway activation and a lack of infiltrating T‐lymphocytes.

## 2 CASE REPORT

**FIGURE 1:**
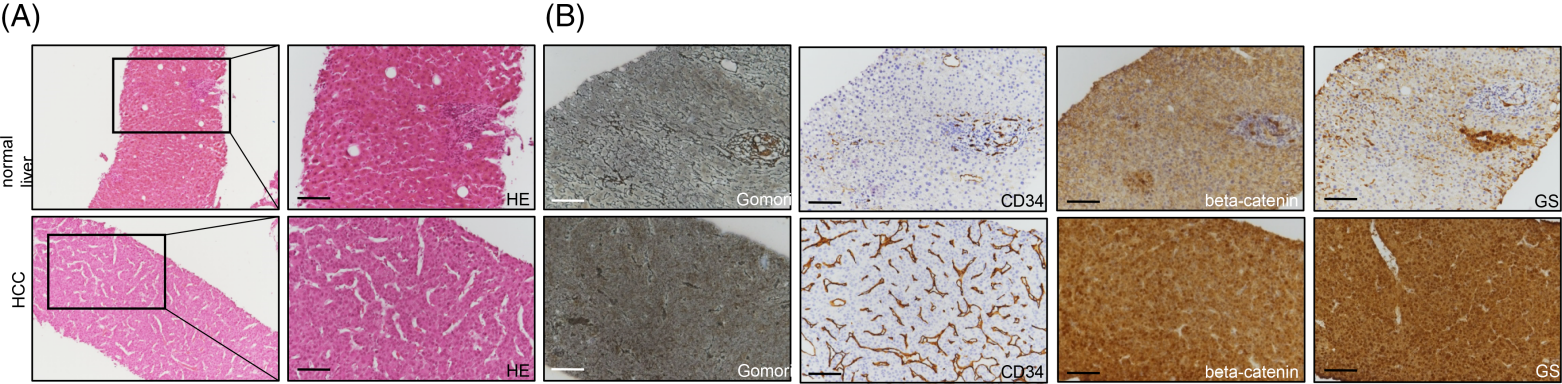
Representative immunohistochemistries of HE displaying the characteristic trabecular growth pattern of the moderately differentiated hepatocellular carcinoma (A). Gomori staining was low in the absence of a liver cirrhosis. CD34 labeled positive endothelial cells in the tumor area in comparison to the normal liver. Glutaminsynthetase (GS) and ß‐catenin revealed a strong staining pattern within the hepatocellular carcinoma

**FIGURE 2:**
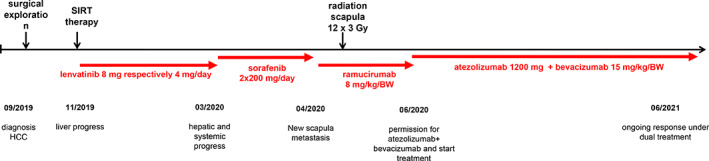
Timeline to illustrate the handling and different treatments of the patient

**FIGURE 3:**
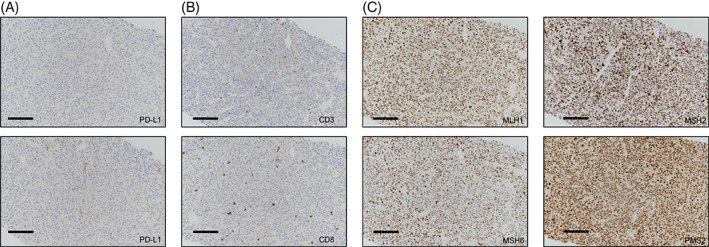
Representative immunohistochemistries of PD‐L1 (A), CD3 and CD8 T‐lymphocytes (B) revealed a low PD‐L1 score and a lack of infiltrating T‐cells in the tumor. Markers for microsatellite instability such as MLH1, MSH2, MSH6, and PMS2 presented a stable expression (C)

**FIGURE 4:**
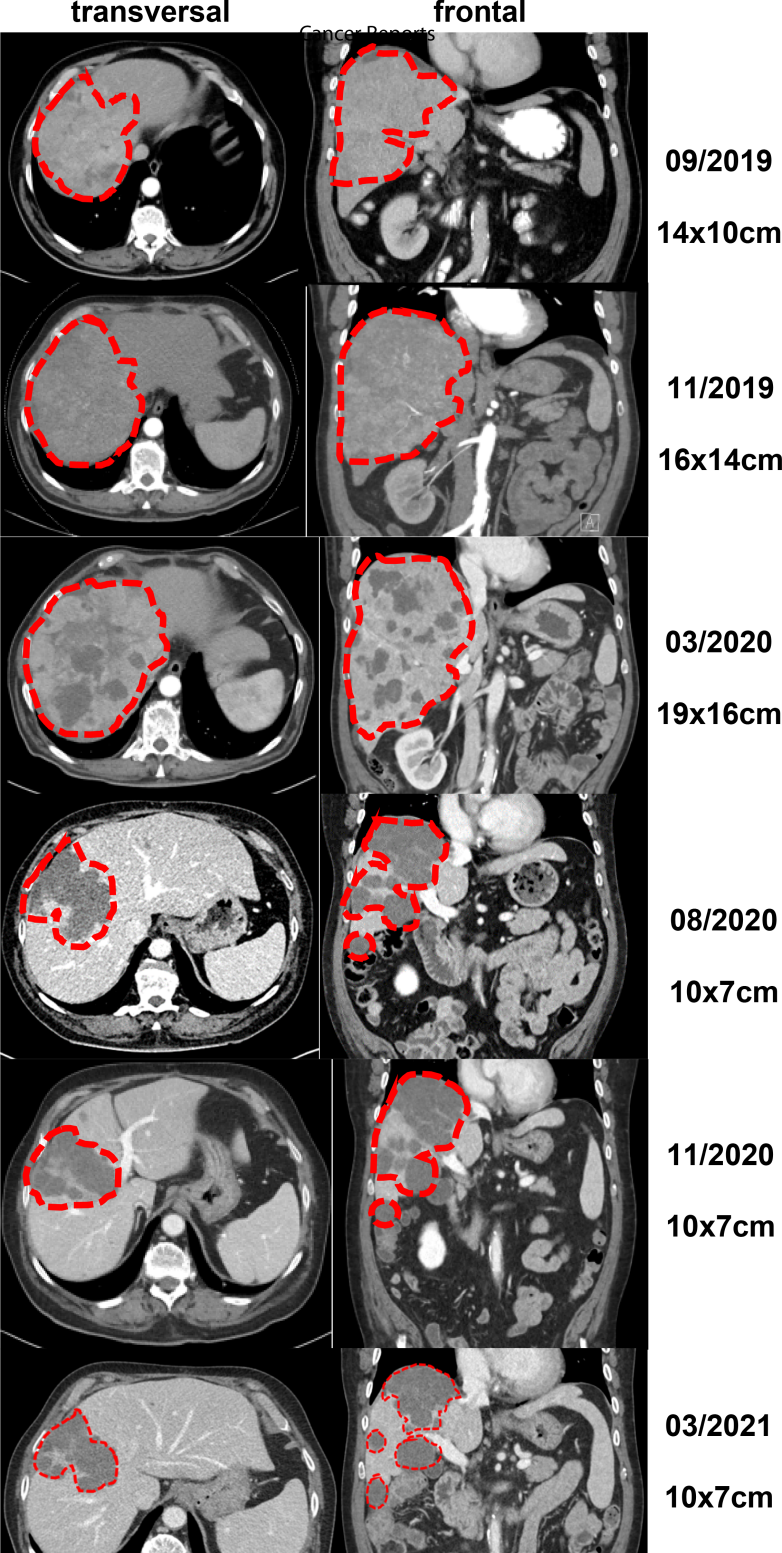
Representative computer tomographic images of the hepatocellular carcinoma in the liver in frontal and transverse views

We diagnosed a male patient (September 2019) with a large solitary liver mass of 14 × 10cm in a noncirrhotic situation. Tumor biopsy presented infiltrates of a moderately differentiated HCC (G2) (Figure 1). Due to nonresectability (proved by surgical exploration) SIRT (selective internal radiotherapy) was initiated and after tumor progress systemic therapy was initiated in November 2019. However, side effects and increasing deterioration of the nutritional status led to several treatment interruptions. After therapy with lenvatinib, sorafenib and ramucirumab (although AFP negative) the primary tumor in the liver was progressive (more than 25 cm in its largest extension) and a new lesion of the left adrenal gland, multiple suspicious abdominal lymph nodes and suspected diffuse small lung metastases occurred (timeline Figure 2). At this point we applied to the health insurance for permission to start a therapy with atezolizumab plus bevacizumab, not approved at that time. In parallel, we evaluated markers for response to immunotherapy of the liver biopsy made during first diagnosis. The immune checkpoint molecule PD‐L1 (TPS = 0% and CPS = 1) was negative (Figure 3(A)). Similarly, there was no significant infiltration of T cells (Figure 3(B)) and the tumor presented a microsatellite stable situation (Figure 3(C)). During workup for immune markers, the tumor and matched normal liver tissue were reexamined. Based on a strong and homogeneous glutaminsynthetase (GS) expression ß‐catenin was assessed and depicted a ubiquitous nuclear staining (Figure 1(B)). Given the literature to the pattern of GS and ß‐catenin, a *CTNNB1*‐exon‐3‐mutation can be suspected. Meanwhile, the scapula metastasis was irradiated with 12 × 3 Gray and after approval by the health insurance, the first cycle of the combination therapy of atezolizumab (1200 mg) and bevacizumab (15 mg/kg/KG) was administered in May 2020. The weight increased continuously from 55 to 82 kg (within 12 weeks) and the only side effect of the combination therapy was hypertension (CTCAE grade 3), which had to be treated with two antihypertensive drugs. After four cycles of therapy with atezolizumab and bevacizumab, a complete liquidation of the previously solid hepatic tumor masses was observed. Radiologically, the findings were characterized as multiple cystic lesions without evidence for vital tumor tissue (Figure 4). The pulmonary and lymphonodal manifestations were also regressive, as was the bone lesion on the scapula. Supportive nutrition and pain therapy were stopped and the patient is currently in an excellent general condition. Therefore, continuation of the therapy with atezolizumab plus bevacizumab is still ongoing every 3 weeks and the excellent clinical and radiological response remains confirmed (state June 2021).

## 3 DISCUSSION

From a scientific point of view there is a strong rationale for combining immunotherapy with VEGF inhibitors. Via complex mechanisms VEGF inhibition can modulate the immunosuppressive TME into an immunostimulatory micromilieu.^3^ The results of the IMbrave study confirmed this preclinical hypothesis for HCC and thus may explain why checkpoint monotherapies have failed in the first‐ and second‐line setting. Therapies with tyrosine kinase inhibitors (TKIs) such as lenvatinib (NCT03713593, LEAP‐002 study) or cabozantinib (NCT03755791, COSMIC‐312 study) may also be very promising in this context; however, the spectrum of side effects of TKIs has to be taken into account in patients with reduced performance status. Antiangiogenic therapy in combination with immunotherapy appears to be independent of the PD‐L1 status. In addition, emerging evidence arises that activated Wnt/β‐catenin signaling is associated with nonimmunogenic “cold” tumors and may be implicated in primary resistance to immunotherapy.^4^ This hypothesis is supported by preclinical and clinical data.^5^ Cells from the innate and adaptive immune response such as Treg cells and polarized M2 macrophages secrete angiogenic factors to promote unrestrained angiogenesis and vascular immaturity.^6^ It is precisely through this state that VEGF inhibition could have a crucial part in the effect of immunotherapy. Bevacizumab may normalize the aberrant vascular‐immune crosstalk by reorganization of malformed tumor vessels to improve the infiltration of CD8+ T and CD4+ TH1 cells into the TME. Therefore, combination therapy with atezolizumab and bevacizumab should be investigated more extensively in the subgroups of HCC patients with *CTNNB1* mutation as well as in noncirrhotic HCC.

## 4 CONCLUSION

To the best of our knowledge, this is the first report showing an impressive response mediated by atezolizumab and bevacizumab in the fourth‐line setting of a metastatic HCC in a patient with noncirrhotic liver and ß‐catenin activation.

**Abbreviations**
HCC: hepatocellular carcinoma
NAFLD: non‐alcoholic fatty liver disease
CT: computed tomography
MRI: magnetic resonance tomography
AFP: alpha‐fetoprotein
HVPG: hepatic venous pressure gradient
ECOG: Eastern Cooperative Oncology Group
SIRT: selective internal radiotherapy
VEGF: Vascular Endothelial Growth Factor

## CONFLICT OF INTEREST

The authors declare that there is no conflict of interest. There was no financial support. Written consent has been obtained from the patient.

## ETHICAL STATEMENT

Written informed consent for publication was obtained from the patient.

## AUTHOR CONTRIBUTIONS

*Conceptualization*: Sebastian Krug and Laura Mattheis. *Data Curation*: Sebastian Krug and Laura Mattheis. *Writing ‐ Original Draft*: Sebastian Krug, Laura Mattheis Jonas Rosendahl and Patrick Michl. *Writing ‐ Review & Editing*: Sebastian Krug, Laura Mattheis, Jonas Rosendahl and Patrick Michl. *Visualization*: Laura Matthei and Sebastian Krug.

## Data Availability

The data that support the findings of this study are available on request from the corresponding author.
